# Systematic review: Advances of fat tissue engineering as bioactive scaffold, bioactive material, and source for adipose-derived mesenchymal stem cells in wound and scar treatment

**DOI:** 10.1186/s13287-021-02397-4

**Published:** 2021-06-02

**Authors:** Pietro Gentile, Aris Sterodimas, Claudio Calabrese, Simone Garcovich

**Affiliations:** 1 Department of Surgical Science, “Tor Vergata” University, Via Courmayeur, 102, 00133 Rome, Italy; 2 Academy of International Regenerative Medicine & Surgery Societies (AIRMESS), 1201 Geneva, Switzerland; 3 Department of Plastic and Reconstructive Surgery, Metropolitan General Hospital, 18547 Athens, Greece; 4 San Rossore Breast Unit, 56122 Pisa, Italy; 5 Institute of Dermatology, F. Policlinico Gemelli IRCSS, Università Cattolica del Sacro Cuore, 00168 Rome, Italy

**Keywords:** Adipose-derived mesenchymal stem cells (AD-MSCs), Stromal vascular fraction (SVF), Fat grafting, Plastic surgery, Regenerative plastic surgery, Wound healing, Scar treatment

## Abstract

**Abstract:**

Fat tissue (FT) has been used for many years in regenerative surgery as a bioactive material through the lipofilling/fat graft (F-GRF)–nano-fat technique, as a bioactive scaffold when it was enriched with adipose-derived mesenchymal stem cells (AD-MSCs) contained in the stromal vascular fraction (SVF), and as a direct source of AD-MSCs used in wound healing (WH) and scar treatment (ST). This systematic review aims to describe the advances in FT engineering applied to regenerative surgery (from bench to clinic), through the use of AD-MSCs, SVF contained in F-GRF in WH and ST. The work has been performed by assessing in the selected studies autologous graft of AD-MSCs, SVF, and F-GRF compared to any control for ST and WH. The protocol was developed following the Preferred Reporting for Items for Systematic Reviews and Meta-Analyses-Protocols (PRISMA-P) guidelines. A multistep search of the PubMed, MEDLINE, Embase, PreMEDLINE, Ebase, CINAHL, PsycINFO, Clinicaltrials.gov, Scopus database, and Cochrane databases has been performed to identify papers on AD-MSCs, SVF, and F-GRF use in WH and ST in which FT was used as bioactive material–scaffold and source of AD-MSCs. Of the 714 articles initially identified, 453 articles focusing on regenerative strategies in WH and ST were selected and, consequently, only 84 articles that apparently related to AD-MSC, SVF, and F-GRF were analyzed. Of these, 61 articles identified as pre-clinical, experimental, and in vitro, and 5 articles identified as a comment and systematic review were excluded. Only 18 original articles which strictly and exclusively focused on autologous AD-MSCs, SVF, and F-GRF in ST and WH were analyzed. The included studies had to match predetermined criteria according to the PICOS (patients, intervention, comparator, outcomes, and study design) approach. The identified studies described microscopic and clinical outcomes in patients treated with AD-MSCs, SVF, and F-GRF. Collected data confirmed the safety and efficacy of FT both as bioactive material–scaffold and source of AD-MSCs in WH and ST without major side effects.

**Graphical abstract:**

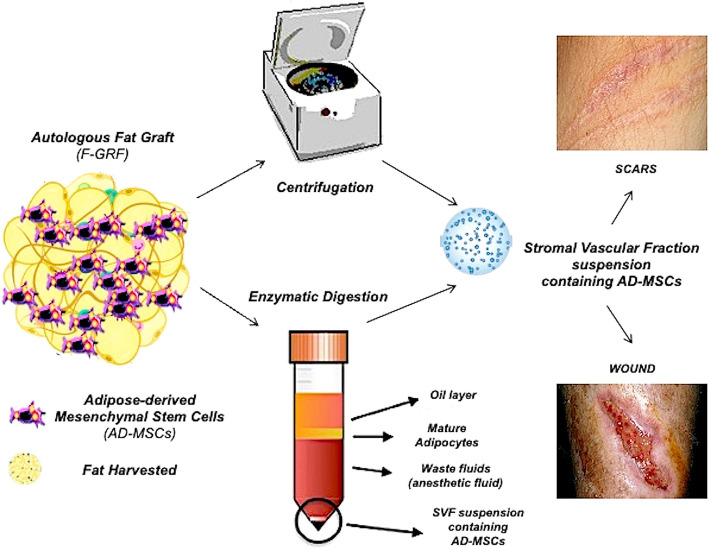

**Supplementary Information:**

The online version contains supplementary material available at 10.1186/s13287-021-02397-4.

## Background

This systematic review provided clinical and microscopic outcomes of the AD-MSCs, SVF, and F-GRF use in WH and ST, analyzing articles in which FT was used as bioactive material through lipofilling/micro-fat/nano-fat/lipostructure techniques, studies in which it was considered only an AD-MSCs’ source, and those in which it was engineered and/or enriched with SVF, acting as a bioactive scaffold for AD-MSCs. Collected data confirmed the safety and efficacy of fat tissue both as bioactive material–scaffold and source of AD-MSCs in WH and ST without major side effects.

## Introduction

A scientific-clinical need exists for the development of biotechnologies to improve wound healing (WH) during scar treatment (ST). The number of investigations evaluating the efficacy of autologous adipose-derived mesenchymal stem cells (AD-MSCs), contained in stromal vascular fraction (SVF) of fat grafting (F-GRF) in WH and ST, has exponentially increased, during the last 15 years (2005–2020). Autologous F-GRF is an interesting procedure in regenerative plastic surgery eagerly used in a growing number of indications, from skin rejuvenation and lipofilling to wound treatment [1]. Most of fat grafts’ regenerative capacity is attributed to AD-MSCs, suspended in a fatty tissue cellular matrix—SVF [2]. It consists of a mixture of endothelial, smooth muscle cells, pericytes, and leukocytes [3]. Thanks to this regenerative activity, the F-GRF, a biological autologous tissue containing several important cellular components (adipocytes and mesenchymal stem cells), extracellular matrix (ECM), vessels, and nerves [3], may be considered both as “bioactive material” when it is grafted in the damaged tissue aiming to improve the scar quality (SQ) and the WH and as “bioactive scaffold” when it is enriched with AD-MSCs. In this last case, F-GRF acts also as a scaffold for the AD-MSCs, representing an autologous biological matrix (cellular and extracellular) in which these cells may be incorporated and transported with the aim to improve the healing time and SQ, via an autologous regenerative approach. The percentage of AD-MSCs in SVF varies depending on the isolation method but is greater than in classic F-GRF [4].

There are two main types of fat tissue’ (FT) processing: mechanical and enzymatic.

Mechanical techniques are appealing because they are simple and quick [5].

Enzymatic SVF isolation is more expensive but can produce higher cell counts and progenitor cell percentage than mechanical methods [6]. In the last 15 years, new approaches to FT processing have emerged. In fact, FT has been used in regenerative plastic surgery as a bioactive material through the lipofilling/micro-fat/nano-fat/lipostructure techniques, as a bioactive scaffold when it was engineered and/or enriched with SVF, and as a direct AD-MSCs’ source. Micro-fat and nano-fat are F-GRF derivates, and they are considered promising methods of lipofilling, especially in the treatment of scars’ interesting superficial skin layers [4, 7]. Several techniques based on centrifugation, emulsification, and filtration procedures have been described to obtain nano-fat and micro-fat [8].

AD-MSCs and SVF cells (SVFs), both contained in the SVF, meet the majority of the International Society for Cellular Therapy’s (ISCT) criteria for mesenchymal stem cells (MSCs). The ISCT, as recently analyzed [8], suggested four parameters to define MSCs:

1. MSCs are disc-adherent in standard cultures;
2. MSCs differentiate in adipocytes, chondroblasts, and osteoblasts;
3. MSCs express CD73, CD90, and CD105; and
4. MSCs do not express CD11b, CD14, CD19, CD34, CD45, CD79, c-kit, and human leukocyte antigen-DR.

The SVF is considered a rich source of AD-MSCs, as each milliliter of adipose tissue offers 300,000 SVFs, of which 1–3% are represented by AD-MSCs (3000–9000/ml) [8]. According to the ISCT and the International Federation for Adipose Therapeutics and Science (IFATS), SVFs and AD-MSCs needed a viability of 70% and 90%, respectively. The percentage of stromal progenitors evaluated with a fibroblast colony-forming unit assay is expected to be 1% for SVFs and 5% for AD-MSCs. The SVFs’ identity, phenotype, is considered closely related to the adipose microenvironment and identified through a typical marker profile. In detail, the SVF immunophenotype should display the following typical marker profile for stromal cells: CD44, CD73, CD13, CD90, CD29 positive (40%), and CD34 positive (20%), but CD31 (20%) and CD45 negative (50%). In contrast, AD-MSCs should be positive for CD29, CD13, CD44, CD90, CD73, and CD105 (>80%), but negative for CD31, CD45, and CD235a (2%).

The aim of regenerative strategies in ST and WH must be the development of new autologous-biotechnologies to involve AD-MSCs and SVF by ex vivo and in vitro culture or by in vivo regeneration and bio-stimulation. Autologous F-GRF as a bioactive material and scaffold has been of great interest for application in soft tissue defects. Moreover, several early efforts in the field focused on isolating AD-MSCs and SVF via enzymatic or mechanical procedures ex vivo for subsequent introduction back into the patient.

However, a major limitation encountered in this area has been the difficulty in obtaining cells to sufficient numbers for human use and the necessity to perform as cell expansion only in Good Manufacturing Practices (GMP) laboratories [8].

SVF and AD-MSCs improve adipogenesis, vascularization, and growth factor (GF) production; hence, some have tried using them in ST [9–11]. The scientific data on this topic has not been properly collected and summarized. Few systematic reviews have been conducted regarding autologous fat transplantation/lipofilling in the treatment of scars, but none has focused also on the use of isolated AD-MSCs/SVF [12–14] or on the use of F-GRF as a bioactive scaffold for them.

In this systematic review, data from investigations reporting the use of AD-MSCs, SVF, and related F-GRF, in ST, analyzing WH, to evaluate such interventions’ efficacy, were gathered.

## Methods

### European rules and international guidelines on AD-MSCs and SVF manipulation

The AD-MSCs and SVF isolation procedures are regulated by the Food and Drug Administration (FDA) and European Medicines Agency (EMA). The European rules are represented by Regulation no. 1394/2007 of the European Parliament for Advanced Therapies (http://www.trovanorme.salute.gov.it/norme/renderNormsanPdf?anno=0&codLeg=47245&parte=1%20&serie=), which introduces the definition of “tissue engineering products” (TEP). Cells and tissues should only be considered TEP if they undergo “significant manipulation.” The same regulation defines the difference between minimal and extensive manipulation.

To complete the description of the rules available in the European legislative panorama and the translation of extracts related to the subject matter, the authors note the “Reflection paper on classification of advanced therapy medicinal products” of May 21, 2015, EMA/CAT/600280/2010 rev.1 Committee for Advanced Therapies (CAT) (http://www.ema.europa.eu/docs/en_GB/document_library/Scientific_guideline/2015/06/WC500187744.pdf).

Briefly, here were reported the Criteria for Somatic Cell Therapy Medicines (sCTMP) and TEP:

1. sCTMP and TEP contain or consist of engineered tissues and/or cells. To be considered “engineered,” tissues or cells must meet at least one of the following criteria:
  - Extensive manipulation (cell culture based on cell expansion, genetic modification of these, their differentiation/stimulation with growth factors (GFs))
  - Various essential functions (not homologous use)

Enzymatic digestion of a tissue to release cells is also considered to be substantial manipulation when the aim is to dissociate cell–cell contacts and the released cells are administered into patients with or without subsequent manipulation. An example would be keratinocytes from the skin, for which enzymatic digestion would destroy the tissue architecture and functional interactions of the cells, which cannot be regained in the cell suspension: this would be considered as substantial manipulation. If the enzymatic digestion leads to isolation of functionally intact tissue units (e.g., pancreatic islets), or there is scientific evidence that the original structural and functional characteristics are maintained, the procedure is not considered substantial manipulation. In case a tissue is treated to remove cells and to be used without any cellular components (e.g., amniotic membrane, bone), the product is not an advanced therapy medicinal product (ATMPs) because it does not contain cells or tissue.

If the number of certain cells (e.g., Mesenchymal stem Cells (MSCs) in fat grafts) is enriched by selection and the processing does not change the characteristics of the cells, this is not considered a substantial manipulation (http://www.ema.europa.eu/docs/en_GB/document_library/Scientific_guideline/2015/06/WC500187744.pdf), chapter 2.2.4 comma a).

For all the above-mentioned reasons, cells or tissues employed for the same essential function, not extensively or substantially manipulated (including fat enzymatic digestion—if the number of AD-MSCs is enriched by selection and the processing does not change the characteristics of the cells), are not considered a substantial manipulation and must not be considered ATMPs.

The present investigation has been developed in agreement with research contract #1467/2017–associate professor contract #13489/2021 between the first investigator, P.G., and the University “Tor Vergata,” Rome, Italy.

### Search strategy and literature screening

This systematic review was registered in the International Prospective Register of Systematic Reviews (PROSPERO, https://www.crd.york.ac.uk/prospero/#myprospero) with ID code number: CRD42021230163.

This systematic review was conducted in accordance with the Preferred Reporting Items for Systematic Reviews (PRISMA) and Meta-Analysis (http://www.prisma-statement.org) [15].

The research was conducted by two investigators (P.G. and S.G.) in accordance with the PRISMA guidelines and the Cochrane handbook [16]. A multistep search of the PubMed, MEDLINE, Embase, PreMEDLINE, Ebase, CINAHL, PsycINFO, Clinicaltrials.gov, Scopus, and Cochrane databases was performed to identify studies, published before December 1, 2020, on WH and ST treatment with AD-MSCs, SVF, and F-GRF, searching without a language or publishing-time restriction.

**Fig. 1:**
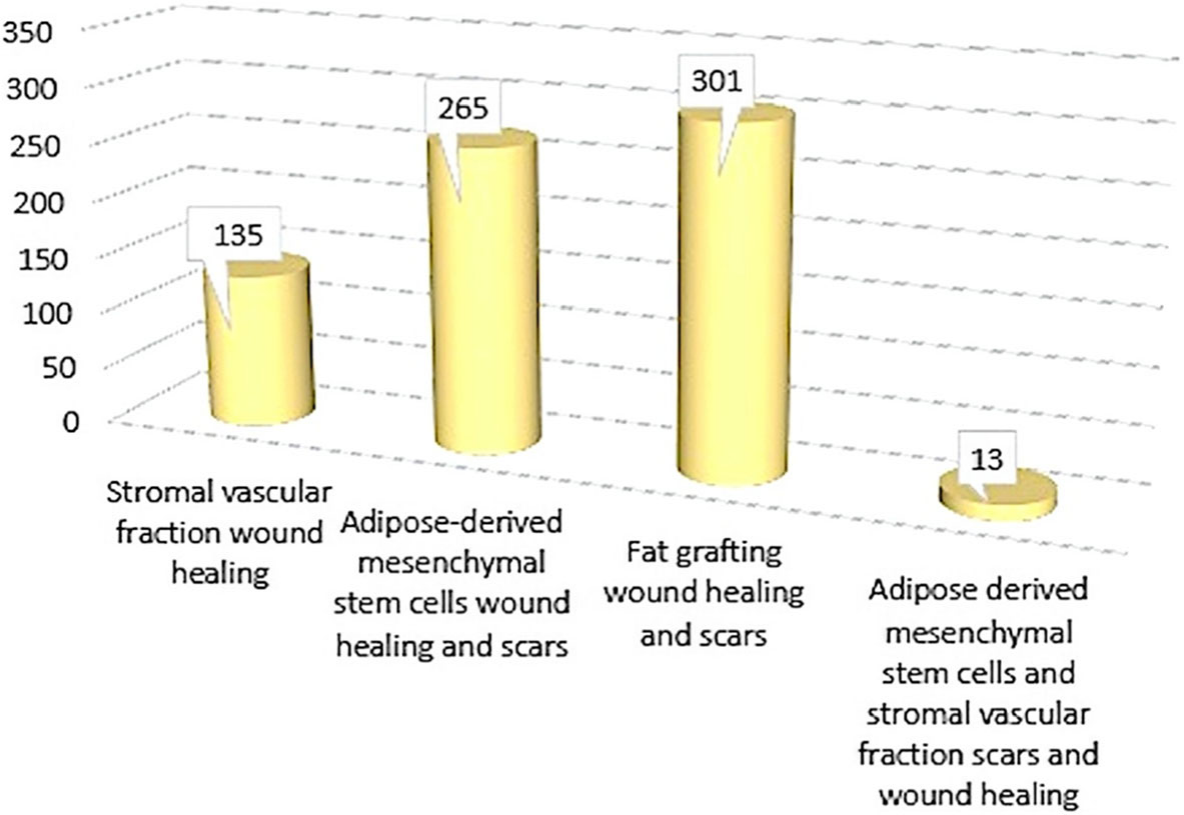
Papers initially found on autologous AD-MSCs, SVF, and F-GRF applications in scar treatment and wound healing

One hundred thirty-five articles using the keyword “stromal vascular fraction wound healing,” 265 articles using the keyword “adipose-derived mesenchymal stem cells wound healing and scars,” 301 using the keyword “fat grafting wound healing and scars,” and 13 articles using the keyword “adipose-derived mesenchymal stem cells and stromal vascular fraction scars and wound healing” were found, as reported in Fig. 1.

### Study assessment

The aim of this systematic review has been to assess the selected articles comparing local injections–grafting of autologous AD-MSCs, SVF, and F-GRF compared to any control for WH and ST.

Articles included in this work had to match predetermined criteria according to the PICOS (patients, intervention, comparator, outcomes, and study design) approach

(https://ro.ecu.edu.au/cgi/viewcontent.cgi?referer=https://www.google.it/&httpsredir=1&article=1010&context=ecupres). Study assessment was based on inclusion and exclusion criteria (Table 1).

**Table 1:** Study assessment based on inclusion and exclusion criteria according to the PICOS (patients, intervention, comparator, outcomes, and study design) approach (https://ro.ecu.edu.au/cgi/viewcontent.cgi?referer=https://www.google.it/&httpsredir=1&article=1010&context=ecupres)

	**Inclusion criteria**
**P**—patients	Age 18–80 years, patients with soft tissue defects, chronic wound, scars, outcomes of scars, acne scars, post-traumatic scars, burns, outcomes of burns
**I**—intervention	Local injection of autologous AD-MSCs, SVF, and F-GRF
**C**—comparator	Any type of control, internal, external, and different product
**O**—outcomes	Healing time, soft tissue volume maintenance, skin quality, scar reduction
**S**—study design	Clinical trial, randomized clinical trial, case-series, case report, case-controlled studies
	**Exclusion criteria**
**P**—patients	Other types of defects and pathologies, patients with platelet disorders, thrombocytopenia, anti-aggregating therapy, use of pharmacological therapeutics targeting WH as advanced dressing, hyaluronic acid, mononuclear cell therapy—platelet-rich plasma was tested as control in PRP studies, bone marrow aplasia, uncompensated diabetes, sepsis, cancer
**I**—intervention	Allogeneic use of AD-MSCs, SVF, and F-GRF, dermal substitute, advanced dressing, hyaluronic acid, steroid injections, surgical procedures
**C**—comparator	Not applied
**O**—outcomes	Not applied
**S**—study design	Expert opinion, comments, letter to the editor, preclinical model (animal studies), in vitro studies, articles identified as bias—not correct match with the keywords used and with the treatment, shorter follow-up than 3 months, review, and systematic review. No limitations were applied on ethnicity or method of fat processing

This systemic review, performed on the PICOS approach, is considered an Evidence-Based Medicine (EBM) 1a level study according to the Oxford Centre for Evidence-Based Medicine (OCEBM), March 2009 (https://www.cebm.net/2009/06/oxford-centre-evidence-based-medicine-levels-evidence-march-2009/).

### Study selection

**Fig. 2:**
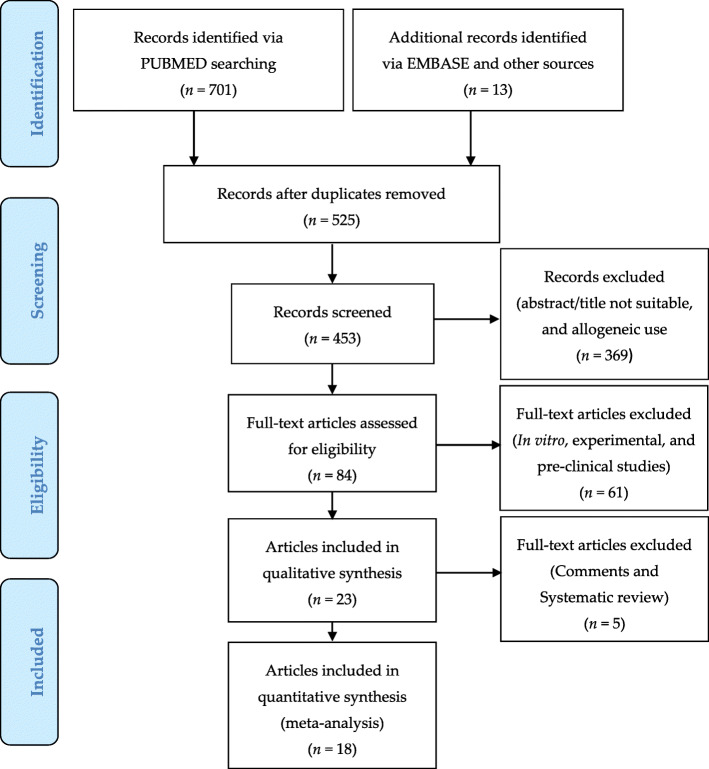
PRISMA flow (Preferred reporting items for systematic review and meta-analysis)

Seven hundred fourteen articles focused on WH and ST were initially identified and selected using PRISMA flow (www.prisma-statement.org) (Fig. 2). A total of 261 articles were excluded. Of this amount, 189 were duplicates and/or not adequate, while 72 articles were considered bias (not correctly match with the treatment and keywords used). Four hundred fifty-three articles were initially selected. Consequently, it was decided to include only clinical trials on autologous use of AD-MSCs, SVF, and F-GRF also referred to as regenerative strategies in WH and ST. For this reason, 369 articles not correctly matched with the topic (abstract/title not suitable, and allogeneic use) were excluded.

Eighty-four articles apparently related to the use of AD-MSCs, SVF, and F-GRF in ST and WH were selected. Of these, 61 articles were identified as pre-clinical, experimental, or in vitro studies. Additionally, of the 23 articles selected, 5 articles were excluded (3 comments and 2 systematic reviews).

Consequently, only 18 articles that strictly and exclusively focused on autologous use of AD-MSCs, SVF, and F-GRF in ST and WH were analyzed and included in this systematic review.

These 18 studies were evaluated and summarized by their study characteristics and study outcomes (Table 2).

**Table 2:** Detailed methodology of selected studies

Authors	Harvest’ procedures	SVF/AD-MSC isolation procedures	SVF yield	Administered solution	Administration route to the recipient site	Cannula/needle	Age of patients	Age of scar(s)	Follow-up period
Wu et al. [17]	**Site**: anterior abdominal **Infiltration**: standard Klein solution **Technique**: 2-mm inner diameter cannula (Mentor BENSAT 330) **Negative pressure** limited to 350 mmHg **Volume**: N/A	1. Lipoaspirate washed three times with saline 2. Gravitational decanting—aqueous phase removed 3. 1000 RCF, 3 min 4. Decantation of oil and aqueous phase 5. Spectroscopy (EP2346989 A1) to isolate SVF-rich fat	N/A	100 + 60 ml SVF. Subsequently 0.5ml autologous serum + 1ml hyaluronic acid (HA) filler; 0.1 PZ-U/ml NB6 collagenase	Subdermal, 100 ml, reverse injections, 3–5 ml per pass layering thin ribbons of graft to reach 20% overcorrection: serum + HA—cross-hatched manner—ring around and underneath the scar sub-dermally; collagenase intradermally	SVF: blunt spatulated tip HA + serum: 22G spinal needle Collagenase: 25G needle	N/A	2 YO	Days 1–5 and 1, 2, 4, 8, and 12 weeks (Ws)
Gentile et al. [18]	N/A	A) SVF-enriched fat 1. Commercial Celution System use 2. Additional wash and centrifugation cycles 3. 5 ml of the enriched fat extracted 4. Added to harvested fat graft B) Coleman’s fat mixed with 0.5 ml of PRP. Fat subjected to centrifugation at 3000 RPM; AFT:PRP = 1ml:0.5ml C) Coleman’s fat	50,000–250,000 nucleated cells per milliliter of fat (automated count–manual count)	Group A: SVF-enriched fat grafts (up to 10ml, of which 5ml of SVF) Group B: 1ml of Coleman’s fat + 0.5 ml PRP Control group: Coleman’s fat	A) Sub-dermally B) Sub-dermally, small tunnels were previously created, cannulas with 1.5-mm diameter	N/A	21–69 YO	N/A	1 y (quantitative data report); mean 60 months (MO)
Carstens et al. [19]	**Site**: N/A **Infiltration**: N/A **Technique**: directly into a sterile processing canister (GID SVF-1) **Negative pressure**: N/A **Volume**: 108 ml dry fat	1. Washed three times with saline 2. 125 ml of lactated Ringer’s + collagenase enzyme 200CDU/ml 3. Incubation—40 min, 38 °C, 150 RPM 4. Centrifugation for 10 min at 800*g* 5. Removal of SVF from the bottom of the device	>4 × 10^7^ mononuclear cells (5 × 10^5^/g of dry fat)	1) 2ml of SVF containing a total of 6 × 106 SVF-derived cells 2) 20ml of SVF enriched with a total of 2.7 × 10^6^ SVF-derived cells	1) Divided and injected into 4 MCP joints 2) Into subcutaneous space of the dorsum of the hand	N/A	58 YO	4 YO	6 Ws; 6, 12, and 24 MO
Elkahky et al. [20]	**Site**: abdomen **Infiltration**: modified Klein’s solution **Technique**: blunt-tipped cannula **Negative pressure**: N/A **Volume**: 250ml	1. Washed 4–6 times with PBS 2. Digestion with 0.2% collagenase at 37 °C for 60 min with agitation 3. DMEM addition to inactivating collagenase 4. Centrifugation at 1500 RPM for 5 min 5. Cellular pellet resuspension in fetal bovine serum and passing through the 100-μm filter to remove debris 6. SVF collection 7. SVF was photoactivated using the AdiLight1 System (Adistem Pty) for 15 min	7 to 86 × 10^6^ cells/sample	SVF resuspended in 10% fetal bovine serum	Intradermal injections	N/A	AT-ASC, 20 to 43 years (mean 26.3 ± 7 years) PRP group, 20 to 44 years (mean 28.7 ± 5 years)	N/A	1 and 3 MO
Zhou et al. [21]	Details N/A, lipo-aspiration from 2 subjects (allogenic)	1. Lipoaspirate digested with 0.75% collagenase type II under gentle agitation for 45 min at 37°C 2. Centrifugation at 300*g* for 10 min 3. Pellet filtered with a 70-μm nylon mesh filter 4. Resuspension in PBS 5. Centrifugation at 840*g* for 10 min 6. Supernatant discarded 7. Cell fraction cultured overnight at 37 °C/5% CO_2_ in culture medium 8. Cell population maintenance for 3–5 days until confluence 9. Medium changed to PBS-free DMEM 10. Exposition to hypoxia (2% O_2_/5% CO_2_ and balanced N_2_) for 72 h 11. Centrifugation at 300*g* for 5 min 12. Filtered using a 0.22-mm syringe filter	N/A	3 ml of ADSC-CM	Topically applied onto fractional laser-treated sites	N/A	24 to 50 years (mean age 36.4)	N/A	1 MO after the last treatment session
Gentile et al. [22]	**Site**: N/A **Infiltration**: N/A **Technique**: Coleman cannula 3-mm diameter with side sharp holes of 1 mm in diameter, luer-lock syringes **Negative pressure**: no high negative pressure (no details) **Volume**: 80ml	A) Classic mechanical emulsification—30 passes B) 160 ml—divided into 1st and 2nd part a. 1st part: i. Automatic filtration (120-μm filter) ii. Centrifugation at 1300 RPM for 10 min iii. SVF pellet collection and SVF suspension filtered (120-μm filter) to final 20 ml b. 2nd part was classically emulsified (30 passes) c. Mixing 1st:2nd 0.2:1 C) Lipoaspirate was centrifuged at 1300 RPM for 10 min, SVF pellet was discarded, and the remaining fat layer was emulsified (30 passes) D) Lipoaspirate slow centrifugation at 80*g* for 3 min with Adipecons in 16 ml stocks, further mechanical emulsification (30 passes)	Mean of 5 samples: A. Classic nano-fat 20,000 ± 3000 B. Supercharged nano-fat 200,000 ± 15,000 C. Centrifuged modified nano-fat 53,334 ± 8000 D. Evo-modified nano-fat 125,000 ± 12,000	A. Classic nano-fat B. Supercharged nano-fat C. Centrifuged modified nano-fat D. Evo-modified nano-fat	Intradermal injections	27G	20–73 YO	N/A	3, 4, 6, and 12 MO
Tenna et al. [23]	**Site**: lower abdomen, flanks, hips, and thighs **Infiltration**: 20 ml of 0.9% NaCl solution, carbocaine 2%, and adrenaline 1/200,000 **Technique**: 3-mm Coleman aspiration cannula **Negative pressure**: manually generated **Volume**: N/A	1. Lipoaspirate centrifuged at 3000 rpm for 3 min 2. Oil removed, and fluid decanted 3. Mechanically emulsified after rinsing, 30 passes through usual connector	N/A	10 ml of nano-fat (7 ml) plus PRP (3 ml)	Sub-cutaneous under deformities	19G cannulas	18 to 52 YO	N/A	1, 3, and 6 MO
Ghareeb et al. [24]	**Site**: preferably abdomen **Infiltration**: 1000 ml of 0.9% saline solution, one ampoule of adrenaline 1 mg/ml, and 15 ml of lidocaine hydrochloride 2% without adrenaline **Technique**: 3-mm cannula with 50-ml syringe **Negative pressure**: manual **Volume**: N/A	Lipoaspirate centrifuged at 3000 RPM for 3 min The purified fat underwent mechanical emulsification (30 passes, through the usual connector)	N/A	Classic nano-fat	Intradermal and subdermal injection of nano-fat	27G sharp needles	8 to 48 YO	N/A	5 days, 2 Ws, 1, 3, and 6 MO
Carstens et al. [25]	**Site**: flanks and abdomen **Infiltration**: N/A **Technique**: N/A **Negative pressure**: N/A **Volume**: 250–350ml	1. Lipoaspirate washed three times with sterile Lactated Ringer’s Solution 2. Collagenase added—200 CDU/ml of total volume, 40 min incubation at 38 °C and 150 rpm 3. Human serum albumin was added (2.5% solution v/v) 4. Centrifuged, 10 min at 800*g* 5. Pellet resuspended in 15 ml Hartmann solution	2.37–9.83 × 10^7^ viable cells/g of fat	SVF	Subcutaneous injection	19G needle	26 ± 6.22 YO	6.7 ± 4.3 YO	6 MO
Bhooshan et al. [26]	**Site**: N/A **Infiltration**: N/A **Technique**: 3-mm mirrored triport Coleman’s cannula **Negative pressure:** manual **Volume:** N/A	Lipoaspirate mechanically emulsified by 30–35 passes through triport connector	N/A	Classic nano-fat	Intralesional	27G needle	32.2 ± 12 YO	3 to 204 MO (17 years); 79.4%—scars <5 years, 20.6%—scars > 5 years	3 months
Gu et al. [27]	**Site**: peri-umbilically **Infiltration**: 20 ml of lidocaine, 0.5%, and 1 ml of 1:1000 epinephrine per 1000 ml of saline **Technique**: 3-mm multi-hole aspiration cannula to a 20-ml syringe **Negative pressure**: manual by a retracted plunger **Volume**: N/A	1. Saline rinsing and filtering 2. Centrifuged at 3000 rpm for 3 min 3. The oil layer was decanted, and the aqueous component drained. 4. For mechanical emulsification, through connected to the Tulip transfer connector with three 1.4-mm holes 30 passes 5. Centrifuged again at 3000 rpm for 3 min	N/A	Condensed nano-fat	Intradermally, after 18G needle is introduced to break underlying adhesions of the scar. Volume restored sub-dermally with fat graft combined with condensed nano-fat through a blunt 1.2-mm cannula	29G needle/1.2 mm blunt cannula	21–62 YO, mean 38.25 YO	3 to 26 years (mean formation time, 7.45 years)	6 months
Lee et al. [28]	**Site**: abdomen **Infiltration**: N/A **Technique**: 3-mm blunt-tipped cannula. Liposuction kit used (Lipokit) **Negative pressure**: N/A **Volume**: 50 ml	1. Centrifugation at 3500 RPM for 4 min 2. Discarding the lower layer (20ml left) 3. Adding collagenase type II and incubation for 30 min in 37 °C (MaxSTEM kit) 4. Centrifugation at 3500 RPM for 3 min 5. Wash in Hartmann solution with 5% dextrose saline and gentamicin 6. Steps 5 and 6 repeated 3 times	5.9 × 10^7^ cells per ml	2 ml of SVF	Subcutaneous and intradermal, no more than 5 ml/case	N/A	Study 1, 14–64 YO (37.47 ± 13.2) Study 2, 19–65 (35.8 ± 14)	Study 1, 3–240 MO (22.3 ± 51.8) Study 2, 0–18 MO (6.53 ± 4.47)	6 MO
Uyulmaz et al. [29]	**Site**: chosen individually **Infiltration**: 900 ml NaCl 0.9%, 0.25 ml adrenaline (1 mg/ml), 20 ml of lidocaine (20 mg/ml) **Technique**: Tonnard 2.4 mm × 20 cm cannula with sharp side 1 mm holes **Negative pressure**: N/A **Volume**: 10–800 ml, mean 165	1. Wash with isotonic saline solution 2. Mechanically emulsification 30 passes (2.4-mm Tulip connector) 3. Filtration through a nylon cloth with 0.5-mm pore size	N/A	Classic nano-fat 1 to 25 ml (mean, 4.6 ml)	Injected intralesionally or intra-dermally	24, 25, or 27G sharp needles	15 to 64 years (mean, 42 years)	15 to 40 years (mean, 5.8 years)	155 ± 49 days (range, 87–312 days)
Abou Eitta et al. [30]	**Site**: abdomen, thighs, buttocks **Infiltration**: modified Klein’s solution **Technique**: blunt-tipped cannula, 60-ml syringe **Negative pressure**: probably manually **Volume**: ~ 50 ml	1. Lipoaspirate washed with PBS and antibiotics/antimycotic 2. Gravitational decanting and discarding of infranatant 3. Steps 2 and 3 repeated 6 times 4. Collagenase type IA and incubation for 37 °C for 1 h 5. Infranatant with SVF aspirated and DMEM with 10% FBS added to inactivate the collagenase 6. Centrifugation at 300*g* for 10 min 7. SVF pellets collected with PBS, filtration with a 100-μm cell strainer 8. Centrifugation at 300*g* for 5 min 9. Optional RBC lysis buffer at room temperature for 5 min; then centrifuged again for 5 min 10. Pellet washed twice with PBS 11. Resuspended in 1ml PBS, ready for injection	Average, 6 × 10^6^ cells	SVF suspended in 1 ml PBS	Reported as injected intradermally (but underneath atrophic scars)	N/A	20 to 45 YO (mean 33.20 ± 6.51)	N/A	1, 2, and 3 MO
Malik et al. [31]	**Site**: abdomen, thighs **Infiltration**: Ringer lactate with epinephrine 1:400,000 **Technique**: liposuction cannula (no details) connected with a 10-ml syringe **Negative pressure**: manually, plunger pulled back only a few ml (?) **Volume**: 25–65 ml, mean 34 ml	1. Gravitational decanting, oil, and aqueous phase discarded 2. 0.075% collagenase added, incubated for 30 min at 37 °C with agitation 3. Centrifugation at 1200 RPM for 5 min 4. Collection of SVF from the pellet	N/A	SVF, 10 ml?	Under scar—subcutaneous	Blunt infiltration cannula	22–45 YO, mean 32.1	N/A	1 and 6 MO
Jan et. Al [32]	**Site**: abdomen, lateral thigh, and/or the gluteal region **Infiltration**: 0.9% saline 1000 ml, lidocaine 30 ml, and 1 ml of 1:1000 epinephrine **Technique**: 3-mm cannula with multiple sharp side holes of 1 mm attached to a 20-ml syringe **Negative pressure**: plunger back by 2 ml **Volume**: N/A	1. Lipoaspirate rinsed with 0.9% saline 2. Emulsification by 30 syringe-syringe passes (unknown connector)	N/A	Classic nano-fat	Selective intradermal or subdermal pre-tunneling. Fanwise pattern with a 1-ml syringe until the skin blanched or yellowish	18G	22.25 ± 5.79 y	>1YO	6 MO
Shalaby et al. [33]	**Site**: preferably abdomen or flanks/inner knees **Infiltration**: 500 ml of 0.9% saline solution, adrenaline 0.5 mg/ml, and 20 ml of lidocaine hydrochloride 2% without adrenaline **Technique**: Unknown cannula, 20-ml syringe **Negative pressure**: retracted plunger **Volume**: N/A	1) Lipoaspirate washed with Lactated Ringer, incubated 3 min 2) Centrifuged at 3000 RPM 3 min 3) Middle layer preserved 4) Mechanically emulsified by shiftings by 30 passes through 2.4-mm tulip connector 5) Another 30 passes with using (1.4-mm tulip connector) 6) 600-μm nanofat filtration	N/A	Nano-fat or nano-fat + PRP	Superficial intradermal nano-fat and additional subdermal injection	28G needle and 22–23G cannula	32.8 ± 11.2 YO (nano-fat + PRP); 26.5 ± 9.1 YO (nano-fat)	6.8 ± 7.6 YO (nano-fat + PRP); 3.3 ± 2.7 (nano-fat)	3 MO
Pallua et al. [34]	**Site**: preferably abdomen and/or others **Infiltration**: adrenaline suspended in saline solution in a ratio of 1:200,000 **Technique**: blunt-tipped thin cannula, with a diameter of 2 mm, and 4 orifices, each gauge measuring 600μm **Negative pressure**: N/A **Volume**: N/A	1. Lipoaspirate is centrifuged at 1200*g* for 3 min 2. The oily and watery layers removed 3. Manual emulsification by 30 passes, usual connector 4. Centrifugation at 1200*g* for 3 min 5. Middle layer preserved	N/A	Condensed nano-fat, in 1 case supplemented with PRP	Various author’s preparation of recipient site before injections. Sub-cutaneous and/or intradermal	27G cannula	41.33 ± 10.53	N/A	6–12 MO

### Data extraction

Data were independently extracted by the first investigator (P.G.) and checked by the last investigator (S.G.) only from the retrieved articles. Any disagreement on the extracted data has been settled by a consensus among P.G. and S.G. including also the co-authors (C.C. and A.S.). The following data have been extracted: first author, year of publication, study design, number of patients, type of procedure, and primary and secondary outcomes. The quality of the included investigations was independently assessed using two investigators (P.G. and S.G.) using the Cochrane Collaboration’s Risk of Bias Assessment tool for randomized controlled trials (RCTs) [16] while using the Newcastle–Ottawa Scale to evaluate the individual non-randomized studies [35].

### Endpoint definition

The efficacy of AD-MSCs, SVF, and F-GRF, in particular of F-GRF/nano-fat as bioactive material–scaffold and AD-MSC source, was primarily evaluated by a reduction in healing time and scar area during WH and by an improvement of soft tissue volume maintenance and skin quality during ST. Secondarily, by the satisfaction of patients from the surveys, and changes of scar’s outcomes compared with pictures and histological analysis taken before and after the treatment sections. Given that, various test methods were taken through the studies included, only the most widely used methods would be set at the endpoints for all pooled studies. All side effects including local injection pain and increased sensitivity in the treated area have been analyzed.

## Results

### Literature search

Seven hundred fourteen articles have been initially identified. Six hundred thirty articles have been excluded for several reasons, including duplicates (*n* = 189), due to incorrect matching after the title’s/abstract’s screening and allogeneic use (*n* = 369), and bias due to incorrect matching with the treatment and keywords (*n* = 72). Eighty-four articles have been initially assessed for eligibility; of this amount, 61 articles considered in vitro, experimental, and pre-clinical studies have been excluded. For the above-mentioned reasons, 23 articles have been selected but only 18 were articles strictly correlated with the autologous use of AD-MSCs, SVF, and F-GRF/nano-fat in ST and WH [17–34].

### Study subjects

Eighteen articles were included in quantitative synthesis (meta-analysis). No significant side effects were reported in the analysis. The human clinical autologous use regarded the treatment of soft tissue defects, scars, acne scars, outcomes of scars, chronic wound, post-traumatic scars, burns, and outcomes of burns.

### AD-MSCs, SVF isolation procedures, and fat graft preparation

The most commonly used procedures were enzymatic SVF isolation [18–21, 25, 28, 30, 31] and nano-fat [22–24, 26, 27, 29, 32–34], while Wu et al. [17] used spectroscopy for SVF isolation. The fatty tissue was harvested from the abdomen or multiple sites, including flanks, hips, or thighs. Protocols of enzymatic SVF isolation varied between studies. Most commonly (6 studies) they included FT digestion with collagenase and subsequent centrifugation. Lee et al. [12, 28] condensed the fat prior to enzyme addition. Zhou et al. [21] cultured AD-MSC fraction after isolation and eventually produced a cell-free medium, rich in GFs and cytokines, later used in the study as the bioactive scaffold. Nano-fat as a bioactive material was used in 9 cases. Its production, however, differed between studies. In six cases, fat condensation was performed prior to mechanical emulsification. In two studies, nano-fat was additionally centrifuged afterward. In three cases, nano-fat was produced classically. Gentile et al. [22] produced 3 modified versions of nano-fat—enriching it with mechanically isolated SVF and performing additional mechanical processing. In this case, the obtained product was a bioactive material–scaffold ready to be injected.

### Selected studies analyzed

The studies analyzed have been represented by case reports (*n* = 3), case series (*n* = 7), case-controlled studies (*n* = 2) [18, 28], and prospective studies (*n* = 7), as analyzed in the supplemental material section detailing.

### Outcomes and endpoints

In addition to clinical evaluation, endpoint evaluation methods included biopsy with immunochemistry stain, photographic evaluation, global photographs, physician global assessment score, and patient global assessment score. The satisfaction surveys and scales were taken from the perspective of patients, and other observers were also used to evaluate the efficacy of AD-MSCs, SVF, and F-GRF/nano-fat in some of the recruited studies.

Eighty-four percent of the studies analyzed (15/18) showed an improvement in soft tissue volume maintenance and skin quality and a reduction of scar area and healing time during ST and WH, when bioactive material–scaffold (FGR-F/nano-fat enriched with PRP/SVF/AD-MSCs–AD-MSCs-conditioned medium) [17–19, 21–23, 34], bioactive material (F-GRF/nano-fat) [24, 26, 27, 29, 32], and AD-MSCs/SVF cellular suspension [20, 25, 28] were used.

### Critical assessment of the study design

Performing a deep analysis of the selected studies during this investigation, a lack of standardized and widely share protocol for the isolation methods of AD-MSCs and SVF has been highlighted, as well as standardized evaluation procedures. In particular, there is a lack of widely shared consensus on the preparation procedures of F-GRF/nano-fat (centrifugation, filtration, emulsification) and on the digestion method (enzymatic vs mechanical). Additionally, the difficulty in clearly interpreting results was determined by the wide range of the studies analyzed (from pilot studies to randomized trials).

### Side effects

No major side effects have been displayed in the analyzed papers. Only tolerable and temporary pain during and immediately after the procedures and transient edema have been described by some patients during fat harvest.

## Discussion

The high number of intervention options to improve WH and scar-tissue quality might cause some confusion during the decision-making process. Dermal substitutes, advanced dressings, hyaluronic acid, fat graft, PRP, CO^2^ and related fractional lasers, steroid injections, dermabrasion, and surgical procedures (like advancement flap, sliding flap, rotation flap, or zeta-plastic) are all important elements in WH and ST procedures [36, 37]. The plastic surgeons should choose an adequate technique according to the clinical examination, promoting the regenerative strategies when possible. In this systematic review, information from 18 selected studies has been harvested, reporting outcomes in a total of 665 patients. This body of evidence is significant, but EBM studies of levels I–II are few. Fifty-five percent of the selected studies (10/18) are case reports or case series. Among RCTs, there was a significant bias risk, represented by the absence of information about allocation concealment, blinded outcome assessment, and selective outcome reporting. Additionally, only small study groups were analyzed among RCTs. Nevertheless, the authors have shown very interesting results, suggesting beneficial effects of AD-MSCs/SVF in ST and related WH. The scars’ and wound healing outcomes obtained with AD-MSCs, SVF, and F-GRF/nano-fat—used as bioactive material and/or bioactive scaffold or as cellular suspension—have been objectively assessed, reducing the risk of bias. Internationally accepted scar quality measuring tools like VSS, POSAS, or VAS enable outcome evaluation [38] and have been used in 50% of the studies (9/18) [21, 24–28, 31–33] reporting a significative clinical improvement after AD-MSCs, nano-fat, and SVF administration. Other investigators also emphasized satisfying results in terms of scar texture, colors, softness, elasticity, vascularization, and hydration after these interventions. SVF was used with success in the treatment of six cases of hand burns by Carstens et al. [19, 25] facilitating the rehabilitation process.

The outcomes analyzed suggest that esthetic results and patients’ satisfaction have been better when AD-MSCs, SVF, or F-GRF/nano-fat enriched with SVFs (bioactive material–scaffold) were administered, in comparison with classic F-GRF. The F-GRF volume maintenance, besides, improves in nano-fat/SVF-treated groups [18, 31]. Three studies compared PRP with AD-MSCs/SVF/F-GRF/nano-fat and suggested improved scar area reduction in the PRP group, in particular when F-GRF was enriched with PRP (bioactive material–scaffold) [18] with little differences in fat resorption, showing clinical outcomes and/or microscopic findings [18, 20, 33].

Abou Eitta et al. [30] prospectively compared SVF with fractional CO^2^ laser in post-acne scar treatment but found no significant differences. Histological analyses undertaken in selected studies showed increased elastin and collagen production, coupled with increased dermal thickness and neovascularization [18, 20–22, 27].

Gu et al. [27] visualized sebaceous and sweat glands, usually absent or scarce in scars, 6 months after nano-fat injection, used as bioactive material.

Zhou et al. [21] showed that topically administered AD-MSC-conditioned medium (bioactive material–scaffold) improves fibers’ alignment. Undoubtedly, the abundance of SVF/AD-MSC isolation methods and multiple nano-fat processing protocols pose a challenge to interpreting collected results. They also reflect a great heterogeneity of clinical practices.

Some authors modified the original nano-fat production procedure by additional centrifugations before and/or after the homogenization step [22–24, 27, 33, 34] improving in some cases the outcomes in terms of cell yield [22].

Gentile et al. [18, 22] showed a post hoc association between AD-MSC quantity and clinical outcomes. The significance of this observation, however, may be undermined by a relatively small study population. In vitro studies demonstrated that similar modifications increase AD-MSC output [33, 38–41] without affecting the composition of secreted proteins [41].

The data outcomes seem to suggest the higher stem cell yield translates into clinical improvement. This concept should be demonstrated through EBM level I–II studies. Factors such as surgeon’s craftsmanship or post-graft care play an important role in shaping the outcome, but they are difficult to assess objectively [42]. This review has been limited by the small group of studies available and analyzed, and the exclusion of pre-clinical studies.

## Conclusions

Analyzed data are substantial and significant, even if with average medical evidence and an EBM level of III and IV (EBM III—case-controlled studies and EBM IV—case series) for beneficial effects of AD-MSCs/F-GRF-related interventions in WH and ST—both clinically and on a microscopic level. Many evidence, suggests that SVF/nano-fat is non-inferior to common approaches, such as PRP or fractional CO^2^ laser in terms of clinical outcomes. Collected data confirmed the safety and efficacy of F-GRF/nano-fat both as bioactive material/bioactive material–scaffold and source of AD-MSCs in WH and ST without major side effects. Given the current treatments differ in methodology and treatment technique, further studies are needed to define standardized protocols. Moreover, large-scale randomized trials still need to be conducted to confirm its efficacy.

For these reasons, the authors invite all the audience to improve the level of publications in this field by focusing prevalently on EBM level I–II studies.

## Supplementary Information

**Additional file 1.** Selected studies analyzed.

## Data Availability

All data generated and/or analyzed during this study are available from the corresponding author upon reasonable request.

## Abbreviations

WH: Wound healing
ST: Scar treatment
SQ: Scar quality
ECM: Extracellular matrix
AD-MSCs: Adipose-derived mesenchymal stem cells
SVF: Stromal vascular fraction
SVFs: Stromal vascular fraction cells
MSCs: Mesenchymal stem cells
GMP: Good Manufacturing Practices
FDA: Food and Drug Administration
EMA: European Medicines Agency
TEP: Tissue engineering products
CAT: Committee for Advanced Therapies
sCTMP: Somatic Cell Therapy Medicines
ATMPs: Advanced Therapy Medicinal Products
EBM: Evidence-Based Medicine
RCTs: Randomized clinical trials
VSS: Vancouver Scar Scale
POSAS: Patient and Observer Scar Assessment Scale
OSAS: Observer scar assessment score
VAS: Visual analog scale
SBSES: Stony Brook Scar Evaluation Scale
DMEM: Dulbecco’s modified Eagle medium
ECCA: Échelle d'évaluation clinique des cicatrices d'acné
TEWL: Trans-epidermal water loss
PRISMA: Preferred Reporting for Items for Systematic Reviews and Meta-Analyses
OCEBM: Oxford Centre for Evidence-Based Medicine
PICOS: Patients, intervention, comparator, outcomes, and study design
AFT: Autologous fat transfer
N/A: Not available
PRP: Platelet-rich plasma
RCF: Relative centrifugal force
RPM: Revolutions per minute
YO: Years old
MO: Months old
Ws: Weeks
HA: Hyaluronic acid
GFs: Growth factors

## References

[1] Liu W, Shi K. Zhu X, et al Adipose tissue-derived stem cells in plastic and reconstructive surgery: a bibliometric study. Aesthetic Plast Surg. 2020; in press. 10.1007/s00266-020-01615-3 31980863

[2] Harasymiak-Krzyzanowska I, Niedojadło A, Karwat J (2013). Adipose tissue-derived stem cells show considerable promise for regenerative medicine applications. Cell Mol Biol Lett..

[3] Wagner W, Wein F, Seckinger A, Frankhauser M, Wirkner U, Krause U, Blake J, Schwager C, Eckstein V, Ansorge W, Ho AD (2005). A. Comparative characteristics of mesenchymal stem cells from human bone marrow, adipose tissue, and umbilical cord blood. Exp Hematol..

[4] Tonnard P, Verpaele A, Peeters G, Hamdi M, Cornelissen M, Declercq H (2013). Nanofat grafting: basic research and clinical applications. Plast Reconstr Surg..

[5] Schäffler A, Büchler C (2007). Concise review: adipose tissue-derived stromal cells--basic and clinical implications for novel cell-based therapies. Stem Cells..

[6] Aronowitz JA, Lockhart RA, Hakakian CS (2015). Mechanical versus enzymatic isolation of stromal vascular fraction cells from adipose tissue. Springerplus..

[7] Nguyen PS, Desouches C, Gay AM (2012). Development of micro-injection as an innovative autologous fat graft technique: the use of adipose tissue as dermal filler. J Plast Reconstr Aesthet Surg..

[8] Gentile P, Calabrese C, De Angelis B (2019). Impact of the different preparation methods to obtain human adipose-derived stromal vascular fraction cells (AD-SVFs) and human adipose-derived mesenchymal stem cells (AD-MSCs): enzymatic digestion versus mechanical centrifugation. Int J Mol Sci..

[9] Han TT, Toutounji S, Amsden BG (2015). Adipose-derived stromal cells mediate in vivo adipogenesis, angiogenesis and inflammation in decellularized adipose tissue bioscaffolds. Biomaterials..

[10] Spiekman M, van Dongen JA, Willemsen JC, Hoppe DL, van der Lei B, Harmsen MC (2017). The power of fat and its adipose-derived stromal cells: emerging concepts for fibrotic scar treatment. J Tissue Eng Regen Med..

[11] Wang J, Liao Y, Xia J, Wang Z, Mo X, Feng J, He Y, Chen X, Li Y, Lu F, Cai J (2019). Mechanical micronization of lipoaspirates for the treatment of hypertrophic scars. Stem Cell Res Ther..

[12] Lee G, Hunter-Smith DJ, Rozen WM (2017). Autologous fat grafting in keloids and hypertrophic scars: a review. Scars Burn Heal..

[13] Riyat H, Touil LL, Briggs M (2017). Autologous fat grafting for scars, healing and pain: a review. Scars Burn Heal..

[14] Crowley C, Lim S-K, To K (2019). Autologous adipose tissue grafting for the management of the painful scar. Cytotherapy..

[15] Moher D, Liberati A, Tetzlaff J (2009). Preferred Reporting Items for Systematic Reviews and Meta-Analyses: the PRISMA statement. PLoS Med.

[16] Higgins JP, Altman DG, Gøtzsche PC (2011). The Cochrane collaboration’s tool for assessing risk of bias in randomized trials. BMJ..

[17] Wu AY, Morrow DM. Autologous fat transfer with in-situ mediation (AIM): a novel and compliant method of adult mesenchymal stem cell therapy. J Transl Med. 2013;11(1):136. 10.1186/1479-5876-11-136 PMC3679930 23725573

[18] Gentile P, De Angelis B, Pasin M (2014). Adipose-derived stromal vascular fraction cells and platelet-rich plasma: basic and clinical evaluation for cell-based therapies in patients with scars on the face. J Craniofac Surg..

[19] Carstens MH, Correa D, Llull R (2015). Subcutaneous reconstruction of hand dorsum and fingers for late sequelae of burn scars using adipose-derived stromal vascular fraction (SVF). CellR4.

[20] Elkahky HO, Fathy G, Abu-Zahra FA, Afify AA (2016). Autologous adipose-derived adult stem cells injection versus platelet-rich plasma injection in the treatment of rolling post-acne scars. J Egypt Women’s Dermatol Soc.

[21] Zhou BR, Zhang T, Bin Jameel AA, Xu Y, Xu Y, Guo SL, Wang Y, Permatasari F, Luo D (2016). The efficacy of conditioned media of adipose-derived stem cells combined with ablative carbon dioxide fractional resurfacing for atrophic acne scars and skin rejuvenation. J Cosmet Laser Ther..

[22] Gentile P, Scioli MG, Bielli A (2017). Comparing different nanofat procedures on scars: role of the stromal vascular fraction and its clinical implications. Reg Med..

[23] Tenna S, Cogliandro A, Barone M, Panasiti V, Tirindelli M, Nobile C, Persichetti P (2017). Comparative study using autologous fat grafts plus platelet-rich plasma with or without fractional CO2 laser resurfacing in treatment of acne scars: analysis of outcomes and satisfaction with FACE-Q. Aesthetic Plast Surg..

[24] Ghareeb F, Elsakka DM, Alkhateep Y (2017). Improving esthetic outcome of facial scars by fat grafting. Menoufia Med J..

[25] Carstens MH, Pérez M, Briceño H (2017). Treatment of late sequelae of burn scar fibrosis with adipose-derived stromal vascular fraction (SVF) cells: a case series. CellR4.

[26] Bhooshan LS, Geetha Devi M, Aniraj R (2018). Autologous emulsified fat injection for rejuvenation of scars: a prospective observational study. Indian J Plast Surg..

[27] Gu ZC, Li YR, Li H (2018). Use of condensed nanofat combined with fat grafts to treat atrophic scars. Jama Facial Plast Surg.

[28] Lee JW, Park SH, Lee SJ, Kim SH, Suh IS, Jeong HS (2018). Clinical impact of highly condensed stromal vascular fraction injection in surgical management of depressed and contracted scars. Aesthetic Plast Surg..

[29] Uyulmaz S, Macedo NS, Rezaeian F (2018). Nanofat grafting for scar treatment and skin quality improvement. Aesthet Surg J..

[30] Abou Eitta RS, Ismail AA, Abdelmaksoud RA, Ghezlan NA, Mehanna RA (2019). Evaluation of autologous adipose-derived stem cells vs. fractional carbon dioxide laser in the treatment of post-acne scars: a split-face study. Int J Dermatol..

[31] Malik P, Gaba S, Ahuja C, Sharma RR, Sharma RK, Khandelwal N (2019). Role of fat graft alone versus enriched fat graft with stromal vascular filtrate in painful amputation stump. Indian J Orthop..

[32] Jan SN, Bashir MM, Khan FA, Hidayat Z, Ansari HH, Sohail M, Bajwa AB, Shami HB, Hanif A, Aziz F, Choudhery MS (2019). Unfiltered nanofat injections rejuvenate postburn scars of face. Ann Plast Surg..

[33] Shalaby ME-S, Ibrahim SMA, Hassanin MNA (2020). Nanofat combined with platelet-rich plasma injection versus nanofat injection alone in the treatment of atrophic scar. Al-Azhar Med J.

[34] Pallua N, Kim BS (2020). Microfat and lipoconcentrate for the treatment of facial scars. Clin Plast Surg..

[35] Wells GA, Shea B, O’Connell D (2014). The Newcastle-Ottawa Scale for assessing the quality of nonrandomised studies in meta-analyses.

[36] Khansa I, Harrison B, Janis JE (2016). Evidence-based scar management: how to improve results with technique and technology. Plast Reconstr Surg..

[37] Goverman J, Mathews K, Goldstein R, Holavanahalli R, Kowalske K, Esselman P, Gibran N, Suman O, Herndon D, Ryan CM, Schneider JC (2017). Adult contractures in burn injury: a burn model system national database study. J Burn Care Res..

[38] Fearmonti R, Bond J, Erdmann D (2010). A review of scar scales and scar measuring devices. Eplasty..

[39] Mashiko T, Wu SH, Feng J, Kanayama K, Kinoshita K, Sunaga A, Narushima M, Yoshimura K (2017). Mechanical micronization of lipoaspirates: squeeze and emulsification techniques. Plast Reconstr Surg..

[40] Pallua N, Grasys J, Kim BS (2018). Enhancement of progenitor cells by two-step centrifugation of emulsified lipoaspirates. Plast Reconstr Surg..

[41] Prantl L, Eigenberger A, Klein S (2020). Shear force processing of lipoaspirates for stem cell enrichment does not affect secretome of human cells detected by mass spectrometry in vitro. Plast Reconstr Surg.

[42] Khouri RK (2018). Discussion: Enhancement of progenitor cells by two-step centrifugation of emulsified lipoaspirates. Plast Reconstr Surg..

