# Supplementary material for: Systematic review: Advances of fat tissue engineering as bioactive scaffold, bioactive material, and source for adipose-derived mesenchymal stem cells in wound and scar treatment
Source: Stem Cell Res Ther. 2021 Jun 2;12:318. doi: 10.1186/s13287-021-02397-4 (PMC8173738; doi:10.1186/s13287-021-02397-4)
Supplement: Supplementary file 1 — Additional file 1. Selected studies analyzed. [file 13287_2021_2397_MOESM1_ESM.docx]

*3.4. Selected studies analyzed*

The analyzed studies have been represented by case reports, case series, case-controlled studies, and prospective studies.

Regarding the case reports, three studies (n=3) were analyzed [18-20]. Wu et al. [18] reported the results obtained in an adherent scar on the lower back and soft tissue reconstruction grafting a bioactive scaffold composed of F-GRF, SVF-enriched fat, collagenase and hyaluronic acid beneath the scar. At 6 weeks, pain was resolved. Three months later, the scar aspect was corrected in 100% and 77% defect correction on ultrasound examination was reported. Carstens et al. [19] treated hand burn scars with isolated SVF administered into joints and SVF-enriched lipofilling as bioactive scaffold to the hand dorsum. Six weeks after treatment the patient regained full range of motion in previously restricted joints – this effect was corroborated at six, twelve and twenty-four months after surgery. Four months after surgery, the ultrasound detected angiogenesis and new blood vessels in the treated areas. Pallua et al. [20] displayed two patients affected by face scars treated with bioactive scaffold here represented by micro-fat, nano-fat and PRP. In both cases, satisfying aesthetic outcomes were achieved.

Regarding the case series, seven studies (n=7) were analyzed [21-27]. Ghareeb et al. [21] used subcutaneous nano-fat injections as bioactive material to treat facial scars. Six months after surgery, all Vancouver scar scale (VSS) components improved, apart from height. Fat resorption occurred in six cases. 76% of treated patients assessed the results as excellent or good. In his second study, Carstens et al. [22] reported 5 cases of patients affected by burn scars localized predominantly on hands, restricting movement, treated with enzymatically isolated SVF. Six months after surgery, the majority of the treated zones improved in terms of pigmentation (78,6%), flexibility (100%), thickness (90,4%), pruritus (94%), pain (100%) and vascularity (33%). Bhooshan et al. [23] used nano-fat as bioactive material to treat 34 patients with different scar types, the majority of them were hypertrophic (82%) and localized on the face (85%). Three months after surgery, 76,5% of treated patients had good aesthetic results, meant as Patient and Observer Scar Assessment Scale (POSAS) score 6-24, while 23,5% had bad results, meant as POSAS >24. All factors evaluated in POSAS improved after treatment, except for scar surface area. 92,6% of patients with scar history <5 years had good aesthetic results, whereas only 14,3% of patients with older scars shared this outcome. Gu et al. [24] grafted condensed nano-fat as bioactive material in 25 scars. Clinical outcomes were evaluated with POSAS preoperatively and after six months. Significant improvement in all POSAS variables, measured by patients and physicians, apart from pain, itching and vascularization were reported. Microscopical examination showed increased melanin average optical density (0,671 vs. 0,844), but no changes in elastic fibres. Moreover, six months after surgery, immunostaining highlighted previously undetectable sebaceous and sweat glands. Lee et al. [25] reported a case series of 17 patients suffering from a total of 19 scars (hypertrophied, depressed, contractile) who received SVF injection alone or in the course of other procedures (scar revision, F-GRF etc.). Observer scar assessment score (OSAS), visual analog scale (VAS), VSS and stony brook scar evaluation scale (SBSES) were used to assess the clinical outcome. Compared to baseline, OSAS and VSS median scores dropped by 5 and 3, respectively, while SBSES and VAS increased by 1 and 2 after six months. Vascularity, pigmentation, hardness, flexibility and pliability were particularly improved. Uyulmaz et al. [26] treated various scars in 40 patients with nano-fat injections as bioactive material. Scar aesthetic improvement was noticeable at 100 days after treatment. Three independent physicians reviewed the outcome as good in 74% of the treated cases and satisfactory in 18% after three months. Jan et al. [27] reported a series of 48 patients with facial scars, treated with nano-fat as bioactive material. At six-months follow-up, POSAS significantly improved in all patient-assessed parameters, compared to baseline. In the observer’s opinion, the overall score was appreciable, but only pliability and pigmentation were significantly improved.

Regarding the case-controlled studies, two studies (n=2) were analyzed [25,28]. The second study by Lee et al. [25] compared two groups of patients who underwent scar revision with (7 patients) or without (8 patients) SVF injection. The median score in OSAS, VSS and VAS improved in both groups. Height for the SBSES and pliability in the VSS were significantly better in the SVF-treated group, compared to the control one. Gentile et al. [28] studied a group of 30 patients with burn or post-traumatic scars. In patients who received SVF-enriched F-GRF or PRP-enriched F-GRF bioactive scaffold, scars maintained their contour and volume in 63% and 69%, respectively, compared with the control group (39%), treated with F-GRF alone, after 1 year. MRI and ultrasound showed lower fat reabsorption in the SVF and PRP groups.

Regarding the prospective studies, seven studies (n=7) were analyzed [29-35]. Elkahky et al. [29] compared enzymatically isolated SVF and PRP treatments in 20 patients with rolling post-acne scars. Mean scar surface reduction percentage, after one month, did not differ between the groups. However, at three months, a higher reduction percentage was shown in the PRP group (80,2%) compared to the SVF group (66,5%). Histological analysis showed an improved remodelling process with higher density and more orderly alignment of collagen and elastin fibres. Epidermal thickness and collagen content improved similarly in both groups. Elastin was more intensively produced after the PRP treatment (40% vs. 30% in the SVF group). Zhou et al. [30] performed a split-face study in 13 patients affected by facial acne scars. Three courses of topically applied AD-MSCs-conditioned medium (bioactive scaffold) combined with CO^2^ fractional laser were used in monthly intervals on one side of the face. Dulbecco's Modified Eagle Medium (DMEM) was applied to the control side. Patients’ satisfaction with the treated side was significantly higher compared to the control side one month after the 3rd treatment (2,35 ± 0,69 vs. 2,08 ± 0,76). The *échelle d'évaluation clinique des cicatrices* *d'acné* (ECCA) score was lower in AD-MSCs-CM side (32,7 ± 18,1) compared to the DMEM side (26,15 ± 19,16) one month after the 3^rd^ treatment, assessed by two blinded evaluators. Melanin index was lower in the first group throughout the study. Biophysical examination showed improved elasticity, trans-epidermal water loss (TEWL) and hydration on the AD-MSCs-CM treated side. Cheek biopsies showed more ample improvement in collagen (49,98% vs. 36,09%) and elastin (37,61% vs. 26,13%) density compared with control. Gentile et al. [31] compared different nano-fat (bioactive material) procedures in 43 patients with burn or post-traumatic scars. Nano-fat modifications included SVF-enrichment and additional mechanical processing steps, producing a bioactive scaffold, called supercharged nano-fat. Clinical outcomes were assessed after six months by patients and operators, by scoring skin quality factors on a scale from 0 to 5. Best results were obtained in the supercharged nano-fat group, followed by evo, centrifuged and classic nano-fat. SVF yields were measured and compared between the groups. The authors associated SVF cell number with clinical improvement. Tenna et al. [32] studied CO^2^ fractional laser addition to nano-fat + PRP treatment (bioactive scaffold) in a group of 30 patients with chronic acne scars. All patients underwent two courses of either treatment (with or without laser) six months apart. Three months after the 2^nd^ treatment, skin thickness improved in the laser-treated group (p=0,007), but not in the control one (*p*=0,12), compared with the pre-operative values. The calculated change in thickness between the pre-and post-op period, however, did not differ between groups. Baseline skin thickness differed between groups (0.532cm in group A and 0.737cm in B). Measurements were taken with ultrasound. Postoperative patients’ quality of life was similar in both groups, evaluated with FACE-Q module. Abou Eitta et al. [33] conducted a split-face study, comparing SVF and CO^2^ fractional laser in the treatment of post-acne scars in 10 patients. Three months after the surgical intervention, scar severity decreased in both groups, as measured by Goodman & Baron scale with no differences between the cohorts. Scar area percentage was reduced after 2 and three months – similarly in both groups. TEWL improved quicker in the SVF-treated group, however, the final TEWL and hydration outcomes were similar on both sides after three months. No difference in patients’ satisfaction was noted. Malik et al. [34] treated 10 patients with painful amputation outcomes both with a bioactive scaffold composed of SVF-enriched F-GRF which is a bioactive material. POSAS decreased after one and six months the treatment similarly in both groups. Compared to the baseline, post-operative MRI scans showed increased fat accumulation over the amputation outcomes in the SVF-treated groups, but not in the control one. Shalaby et al. [35] compared bioactive material nano-fat (control group) with bioactive material - scaffold nano-fat + PRP (study group) in 60 patients suffering from atrophic scars. Total VSS decreased in the control group by means of 3.3 and in the study group by means of 2.2, with pliability and height improving in both. Results did not differ between cohorts.
